# Veratrum Viride in Cases of Children

**Published:** 1860-05

**Authors:** 


*Veratrum Viride in cases of Children.*—The propriety of using "tinct. veratrum viride," in cases of children under two years old, is a question that has long been mooted by professional characters; and at the present day, at least in this section of South-western Georgia, when the action of that medicine is urgently and strenuously called for, the parents tell you, that Dr. so and so, says it is certain death, when given to children, indiscriminate of age.

Let their assertions be as they may, practical experience has proven to the contrary.

For the past few years I have had numerous cases among children, with which to contend. Some of them were of a highly inflammatory type, with the pulse ranging from 150 to 160, and the most certain remedy I have ever tried in controling the excited circulation, was the "tinct- veratrum viride."

It did not only quell the agitated pulse, but it most generally checked the disease in its very commencement.

I have had considerable experience in the administration of "tinct. digitalis" in inflammatory diseases, with very beneficial results, but yet, I cannot speak with as much confidence of the latter medicine, as I can of the former.

In the administration of "tinct. veratrum viride," there is a peculiarity that I have not seen noticed by any of our writers. It is this: when the pulse of an infant ranges from 150 to 160 beats to the minute, and the "veratrum" is administered in doses according to the age and idiosyncrasies, very frequently the pulse does not lessen in frequency; the patient becomes pale with flabby muscles, and with a profuse perspiration. It is then the inexperienced becomes alarmed, and believes his patient his tending to fatal collapse. And just so would it be, were not the proper counteracting remedies given. While you will observe all these dangerous symptoms supervening, pulse quick, &c., you will discover that its volume is measurably lessened, and now is your time to administer some stimulant, such as brandy, or syrup of ginger; I prefer the brandy. Give it in small doses, at intervals of from three to five minutes, until you perceive a change in the pulse.

Under such treatment you will find the pulse lessening in frequency and increasing in volume, fever subsiding, all symptoms assuming a favorable tendency, and your patient getting better. I have experienced this beneficial action so often, that I rely with great confidence on the use of "tinct. veratrum viride," in cases of children suffering from inflammatory affections, more especially pneumonia.—*Oglethorpe Med. & Surg. Journal.* 1860

---

*Illegitimacy in Scotland.*—This, it appears, increases gradually as we proceed northwards; and further, the proportion of illegitimate births in the country districts is considerably higher than in the town districts. In the 125 town districts, 1166 of the births were illegitimate, while in the country districts they numbered 1195; giving the proportion of only 8 per cent. of the births as illegitimate in the town districts, against 10.3 per cent. in the country districts.—*Med. Times and Gaz.*, March 10, 1860.

---

*Holopathy.*—A distinguished physician of Paris, M. Marthal de Calvi, is now lecturing on a new medical doctrine, to which he has given the name of holopathy (holes, *entire;* pathos, *disease.*) M. Marshal considers that diseases, as they come before the medical practitioner, are only phases or episodes of a general affection of the organism, which affection or diathesis produces the episodes when circumstances favor their appearance. The lectures are creating some sensation in the French capital.—*Lancet,* March 10.

